# Parenting Stress and Mental Health in Midlife Adults: Evaluating the Role of Gender

**DOI:** 10.1093/geroni/igab046.3123

**Published:** 2021-12-17

**Authors:** Jean Choi, Elizabeth Munoz, Robin Corley, Sally Wadsworth, Chandra Reynolds

**Affiliations:** 1 The University of Texas at Austin, Austin, Texas, United States; 2 The University of Texas at Austin, The University of Texas at Austin, Texas, United States; 3 University of Colorado Boulder, Boulder, Colorado, United States; 4 University of California Riverside, Riverside, California, United States

## Abstract

Parenthood is a major source of stress in midlife that can have adverse consequences for long-term mental health trajectories. Yet, little research asks how parenting stress impacts mental health for both mothers and fathers in midlife. The current study examined (a) whether parenting stress was associated with parental depressive and anxiety symptoms and (b) whether these associations vary by gender. We utilized data from the ongoing Colorado Adoption/Twin Study of Lifespan behavioral development and cognitive aging (CATSLife); participants were aged 28 to 49 who reported having child(ren) (N = 520). Participants completed surveys that encompassed measures of demographics, relationships, health, and well-being. Overall, multilevel models accounting for non-independence among siblings and with relevant covariates (e.g., number of children, marital status) showed that higher levels of parenting stress were associated with greater depressive (b = .47 (.12), p<.001) and anxiety (b = .27 (.09), p<.05) symptoms. An evaluation of the individual parenting stress items indicated that feeling less happy and more overwhelmed in the parental role were significantly associated with higher levels of anxiety and depressive symptoms. Parents who reported feeling less close to their children were also significantly more likely to report greater levels of depressive symptoms. These effects were consistent across mothers and fathers. Our study provides further insight into the negative associations between parenting stress and mental health among both mothers and fathers, and warrants further investigation into resources that may buffer these negative effects prior to late life.

